# A QS21+ CpG-Adjuvanted Rabies Virus G Subunit Vaccine Elicits Superior Humoral and Moderate Cellular Immunity

**DOI:** 10.3390/vaccines13080887

**Published:** 2025-08-21

**Authors:** Han Cao, Hui Li, Wenzhi Liu, Ning Luan, Jingping Hu, Meijun Kong, Jie Song, Cunbao Liu

**Affiliations:** 1 Yunnan Key Laboratory of Vaccine Research and Development on Severe Infectious Diseases, Institute of Medical Biology, Chinese Academy of Medical Science and Peking Union Medical College, Kunming 650118, China; caohan@imbcams.com.cn (H.C.); huiziyyuu@imbcams.com.cn (H.L.); s2024018008@student.pumc.edu.cn (W.L.); luanning@imbcams.com.cn (N.L.); hujingping@student.pumc.edu.cn (J.H.); kmj@imbcams.com.cn (M.K.); 2 NMPA Key Laboratory for Quality Control and Evaluation of Vaccines and Biological Products, Institute of Medical Biology, Chinese Academy of Medical Science and Peking Union Medical College, Kunming 650108, China

**Keywords:** rabies virus Glycoprotein (RABV-G), QS21, CpG, subunit vaccine

## Abstract

Background: Rabies remains a fatal zoonotic disease caused by rabies virus (RABV), posing substantial global health challenges. Current vaccine production faces challenges in manufacturing efficiency and cost-effectiveness. The RABV glycoprotein (RABV-G) serves as the key antigen for eliciting protective immunity. Methods: We developed a novel QS21+CpG-adjuvanted RABV-G subunit vaccine and systematically compared its performance against three control formulations: mRNA vaccine composed of H270P-targeted mutation packaged in lipid nanoparticles (LNP), named LNP-mRNA-G-H270P, commercial inactivated vaccine, and alum-adjuvanted RABV-G subunit vaccine. Results: The result show that the G+QS21+CpG subunit vaccine elicited superior humoral immunity, as evidenced by significantly higher RABV-G-specific IgG titers and virus-neutralizing antibody responses compared to all other groups. The LNP-mRNA-G-H270P vaccine maintained its expected cellular immunity advantage, with the G+QS21+CpG group exhibiting moderately reduced but still significant levels of IFN-γ-secreting splenocytes and levels of IL-2 in the supernatant of spleen cells, as well as IFN-γ-producing CD4+ T cells. Both LNP-mRNA-G-H270P and G+QS21+CpG vaccine groups provided 100% protection against lethal challenge (50LD_50_ RABV). Conclusions: These findings provide novel vaccine/adjuvant strategies for rabies while elucidating platform-specific immunogenicity patterns, offering critical insights for pathogens requiring balanced humoral/cellular immunity.

## 1. Introduction

Rabies caused by rabies virus (RABV) genotype 1 is one of the most common fatal infections worldwide, predominantly in Asia and Africa, where limited access to timely post-exposure prophylaxis (PEP) exacerbates mortality rates [1,2]. RABV infiltrates the central nervous system, leading to fatal encephalomyelitis once clinical symptoms manifest, underscoring the critical need for effective prophylactic interventions [3]. Historically, rabies vaccines have evolved from Louis Pasteur’s pioneering inactivated nerve tissue vaccines to modern cell-culture-derived inactivated vaccines (e.g., human diploid cell vaccine, Vero cell vaccine), which remain the gold standard for PEP [4]. However, these vaccines require multiple intramuscular doses over weeks, incur high production costs, and exhibit suboptimal immunogenicity in resource-limited settings, where cold chain infrastructure and healthcare accessibility are often inadequate [5].

The emergence of mRNA vaccines has revolutionized vaccine development, offering rapid production and potent immune responses. For instance, an mRNA vaccine encoding a pre-fusion-stabilized RABV glycoprotein (RABV-G) with a H270P mutation demonstrated robust humoral and cellular immunity in mice, surpassing commercial inactivated vaccine [6]. Subunit vaccines, however, rely heavily on adjuvants to mimic the immune-stimulating signal of live or inactivated viruses. Traditional adjuvants like aluminum salts (e.g., alum) enhance humoral responses but postpone the production of rabies virus neutralizing antibody (RVNA) [7]. In contrast, toll-like receptor (TLR) agonists, such as CpG and QS21, offer mechanistic advantages: CpG activates TLR9 on plasmacytoid dendritic cells, driving Th1 polarization and IFN-γ secretion, while QS21, a saponin-derived adjuvant, enhances antibody responses and promotes specific T-cell responses [2,8]. In fact, CpG and QS21, either used alone or in combination with other adjuvants, have demonstrated remarkable efficacy in potentiating the immunogenicity of subunit vaccines against a broad spectrum of pathogens. Specifically, CpG has been shown to enhance the immune responses elicited by subunit vaccines against the Severe Acute Respiratory Syndrome Coronavirus 2 (SARS-CoV-2), Respiratory Syncytial Virus (RSV), and Enterovirus 71 (EV71) [9,10,11,12], while QS21-adjuvanted formulations for SARS-CoV-2, influenza, herpes zoster, and RSV have been reported to induce more robust polyclonal antibody responses and neutralizing titers [13,14,15].

It is reported that the synergistic combination of these two adjuvants could address the limitations of single-component systems, potentially accelerating antibody development and enhancing both humoral and cellular immunity which has been reflected in the development and application of certain subunit vaccines [16,17]. In this study, we innovatively develop a subunit vaccine comprising recombinant RABV-G co-administered with a QS21+CpG adjuvant system (G+QS21+CpG group) and compare the cellular and humoral immune response with the mRNA vaccine (LNP-mRNA-G-H270P), which incorporates the H270P-targeted mutation and is encapsulated in lipid nanoparticles (LNPs), alum-adjuvanted subunit vaccine and commercially available inactivated vaccine in Balb/c mice.

## 2. Materials and Methods

### 2.1. Vaccine Preparation

Four distinct vaccine formulations were prepared for immunization studies (Table 1). The LNP-mRNA-G-H270P vaccine was prepared by a modified procedure as described previously [6]. The critical physicochemical characteristics of the LNP-mRNA-G-H270P formulation were rigorously evaluated during preparation, including LNP particle sizes, polydispersity index (PDI), the integrity of mRNA, and the encapsulation efficiency (Figure S1). For subunit protein vaccines, the G+QS21+CpG formulation was prepared by mixing purified RABV-G (5 μg/dose) with the adjuvant QS21 (5 μg/dose) and CpG oligodeoxynucleotide (10 μg/dose) in sterile phosphate-buffered saline (PBS) immediately prior to administration. Similarly, the G+Alum vaccine was prepared using an antigen adsorption process. Specifically, RABV-G (5 μg/dose) was mixed with aluminum hydroxide adjuvant (1 mg alum/dose) via continuous rotation at 4 °C for 12 h to ensure proper antigen adsorption. As a control, sterile PBS was used in equivalent volumes. All vaccine preparations were stored at 4 °C and administered within 2 h of preparation to maintain stability and biological activity. In addition, the dosage we used in this study is based on previous research experience [16,17,18,19,20].

### 2.2. Animals and Immunization

To investigate the immunogenicity of the experimental vaccine, we conducted studies using a standardized animal model. Healthy female Balb/c mice (4-week-old, weighing 14–16 g) were maintained under pathogen-free (SPF) conditions in the Laboratory Animal Research Facility at the Institute of Medical Biology, Chinese Academy of Medical Sciences (IMBCAMS). Following acclimatization, the animals were randomly divided into five experimental groups (*n* = 15 per group). One of the groups served as the positive control and was administered an optimal dose (1/6 dose) of a commercially available inactivated rabies vaccine preparation (Ningbo Rong’an Biopharmaceutical Co., Ltd., Ningbo, China).

**Figure 1:**
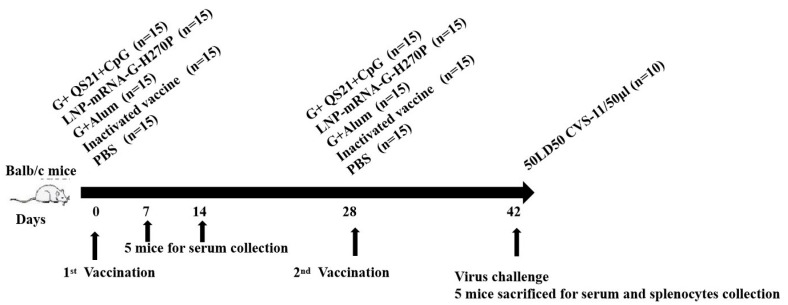
**Vaccine immunization and virus challenge strategy.** Groups of mice (*n* = 15/group) were immunized via intramuscular injection with 50 µL of different antigen formulations, including LNP-mRNA-G-H270P group, G+QS21+CpG vaccine group, G+Alum vaccine group, inactivated vaccine group and PBS group (as a control). Serum was collected at 7, 14 and 42 days after the first vaccination. The virus challenge experiment was conducted 14 days after the final immunization.

The negative control group was administered an equal volume of PBS. Another three additional experimental groups were intramuscularly injected with 50 µL of different antigens including LNP-mRNA-G-H270P vaccine group, G+QS21+CpG vaccine group and G+Alum vaccine group. Mice received an equivalent booster dose 28 days following primary immunization. On the 7th and 14th days, five mice were randomly selected for tail vein blood collection. After 42 days, five mice were randomly selected for enucleation and blood collection, and sacrificed for spleen cells collection. The remaining 10 mice were used for the challenge experiment, and CVS-11 strain was administered via intracranial injection (Figure 1). Blood was collected to measure antibody titers (7, 14 and 42 days) and neutralizing antibody levels (42 days). The spleen cells were harvested for cellular immune analysis (42 days). This study was conducted in strict accordance with institutional and provincial animal welfare regulations. All experimental procedures involving animals received formal approval from the IMBCAMS Animal Care and Use Committee (Ethical Approval No. DWSP202405005) and were performed following the standardized guidelines established by the Yunnan Provincial Laboratory Animal Administration.

### 2.3. Determination of Antibody Titers by Enzyme-Linked Immunosorbent Assay (ELISA)

Serum samples from immunized mice were analyzed for RABV-G-specific antibody levels using an indirect ELISA. Briefly, recombinant RABV-G protein (AtaGenix Laboratories Co., Ltd., Wuhan, China; Cat. No. EVV03501), produced in mammalian cells with >90% purity (confirmed by SDS-PAGE) and exhibiting a monomeric form (predicted molecular weight: 60.01 kDa), was diluted in PBS to a final concentration of 2 μg/mL and used to coat 96-well microplates. After overnight incubation at 4 °C, unbound antigen was removed by washing three times with PBST (PBS containing 0.05% (*v*/*v*) Tween-20). Non-specific binding sites were blocked with 5% (*w*/*v*) skim milk in PBS for 1 h at room temperature. Serial two-fold dilutions of mouse sera (ranging from 1:2000 to 1:4,096,000) were applied and incubated for 1 h at 37 °C. Horseradish peroxidase (HRP)-conjugated goat anti-mouse IgG (Bio-Rad, Hercules, CA, USA; 1:10,000 dilution) was used as the secondary antibody. Following incubation, 3,3′,5,5′-tetramethylbenzidine (TMB; BD, CA, USA) substrate was added, and the enzymatic reaction was terminated after 5 min with 2 mol/L sulfuric acid. Absorbance at 450 nm (OD450) was measured using a microplate reader (BioTek Instruments, Inc., Winooski, VT, USA). Antibody titers were determined based on the highest serum dilution yielding an OD450 value ≥ 0.15 (cutoff threshold). Samples with OD450 values below the cutoff at the initial dilution (1:2000) were assigned a titer of 100 for statistical analysis.

### 2.4. Quantification of Rabies Virus-Neutralizing Antibodies Using Rapid Fluorescent Focus Inhibition Test (RFFIT)

The neutralizing antibody response against rabies virus was evaluated in immunized mouse sera using the RFFIT, performed according to WHO standard protocols [21]. Briefly, serum samples were first heat-inactivated at 56 °C for 30 min prior to analysis. Human rabies immunoglobulin (HRIG; Lot No. 201306, NIFDC National Reference) served as the reference standard for quantification. For the assay, the challenge virus standard (CVS-11 strain, ATCC VR 959) was titrated to 10^6^ fluorescent focus units (FFU)/mL. BSR cells (passages 107-117, IMBCAMS) were prepared at a density of 1 × 10^6^ cells/well. Serum samples were subjected to three-fold serial dilutions (ranging from 1:3 to 1:59,049) in 96-well plates. Each diluted serum sample was then mixed with an equal volume of viral suspension and incubated at 37 °C with 5% CO_2_ for 90 min to allow virus neutralization. Following the neutralization step, BSR cells were added to each well and cultured under the same conditions for 24 h. After incubation, cells were washed with PBS and fixed with 80% cold acetone. Immunofluorescence staining was performed using FITC-conjugated anti-rabies monoclonal antibody (Fujirebio Diagnostics, Malvern, PA, USA; 1:50 dilution in PBS) at 37 °C for 30 min. After thorough washing with PBS, fluorescent foci were examined and quantified using a Leica DMI8 fluorescence microscope. To ensure reliability, all experiments were conducted in duplicate with independent scoring by two investigators. Neutralizing antibody titers, expressed in IU/mL and rounded to two decimal places, were calculated using the Reed and Muench method [22]. This standardized approach allowed for accurate comparison of neutralizing antibody levels across different serum samples.

### 2.5. Enzyme-Linked Immunospot Assay (ELISPOT) of Splenocytes

To obtain single-cell suspensions, spleen tissues were gently homogenized through a 40 µm nylon mesh (BD Biosciences, USA). Erythrocyte depletion was performed by incubating the cell suspension with ammonium–chloride–potassium (ACK) lysing buffer (5 min, RT). Following centrifugation, the purified splenocytes were adjusted to 3 × 10^6^ cells/mL in Roswell Park Memorial Institute (RPMI)-1640 complete medium 10% fetal bovine serum (FBS; Biological Industries, Haemek, Israel) containing 1% penicillin–streptomycin (Thermo Fisher Scientific, Waltham, MA, USA). For ELISPOT assays, cell suspensions were aliquoted at 100 µL/well into 96-well microplates (Corning Inc., Corning, NY, USA).

The ELISPOT assay was performed using a commercial kit (BD Biosciences, USA; catalog no. 551,076 for IL-2 and 551,083 for IFN-γ) according to the manufacturer’s instructions. To assess RABV-G-specific T-cell responses, splenocytes were stimulated with 20 µg/mL of recombinant RABV-G protein (AtaGenix Laboratories Co., Ltd., Wuhan, China; catalog no. EVV03501) and incubated overnight. Following immunostaining, spot formation was quantified using an automated ELISPOT reader system (Autoimmun Diagnostika GmbH, Straßberg, Germany).

### 2.6. Cytokine Analysis

Splenocytes were adjusted to a concentration of 1 × 10^7^ cells/mL using complete RPMI 1640 medium supplemented with 10% FBS and penicillin–streptomycin (both from Biological Industries, Israel). The cell suspension was then plated in 96-well culture plates (Corning Inc., NY, USA) at 100 µL per well. In parallel, positive control wells were treated with PMA (500 ng/mL) and ionomycin (10 µg/mL) (DAKEWE, Beijing, China) at a final volume of 10 µL per well to stimulate cell activation. Experimental wells received protein G stimulation at 10 µg/mL. Following 24 h incubation at 37 °C with 5% CO_2_, culture supernatants were harvested for cytokine quantification. To quantify cytokine levels, 96-well microplates were initially coated with PBS-diluted capture antibodies (anti-IL-2: 3 µg/mL; anti-IFN-γ: 4 µg/mL; Invitrogen, Carlsbad, CA, USA) through overnight incubation at 4 °C. Subsequently, non-specific binding sites were saturated by treatment with 5% (*w*/*v*) skim milk solution at 37 °C for 60 min prior to sample addition.

Supernatant samples (50 µL/well) were incubated for 3 h at room temperature alongside standard curves generated using recombinant mouse IL-2 and IFN-γ (PeproTech, Cranbury, NJ, USA). Subsequent detection employed biotinylated anti-IL-2 or anti-IFN-γ antibodies (2 µg/mL; Invitrogen) followed by HRP-conjugated streptavidin (1 µg/mL; BioLegend, CA, USA), with 1.5 h incubations for each step. Cytokine concentrations were determined by ELISA as previously described.

### 2.7. Flow Cytometry

Flow cytometry reagents and antibodies were procured from BioLegend (San Diego, CA, USA). Splenic single-cell suspensions (1 × 10^7^ cells/mL) were prepared and seeded in 24-well culture plates (2 × 10^6^ cells/well). Following 2 h incubation at 37 °C with 5% CO_2_, cells were stimulated with 10 µg/mL recombinant protein G.

Subsequently, brefeldin A was introduced to inhibit extracellular cytokine secretion, followed by an additional overnight incubation. Following a 16 h incubation period, cell suspensions were collected and assessed for viability with Zombie NIR™ fluorescent dye (BioLegend). Subsequent processing involved fixation and permeabilization steps, followed by intracellular staining with fluorochrome-labeled antibodies targeting murine cytokines: PE-conjugated IFN-γ and APC-conjugated IL-2. Fluorescence data were acquired using a CytoFLEX flow cytometer (Beckman Coulter, Indianapolis, IN, USA) and analyzed with FlowJo software (v10, BD Biosciences, Franklin Lakes, NJ, USA).

### 2.8. Statistical Analysis

Experimental results are presented as mean ± SD values per group. Intergroup comparisons were conducted through one-way ANOVA followed by Dunnett’s multiple comparison test to assess statistical significance, using the G+QS21+CpG vaccine group as the reference control. A two-tailed *p*-value < 0.05 was considered statistically significant.

## 3. Results

### 3.1. QS21+CpG Adjuvanted Vaccine Elicits Superior Humoral Immunity

**Figure 2:**
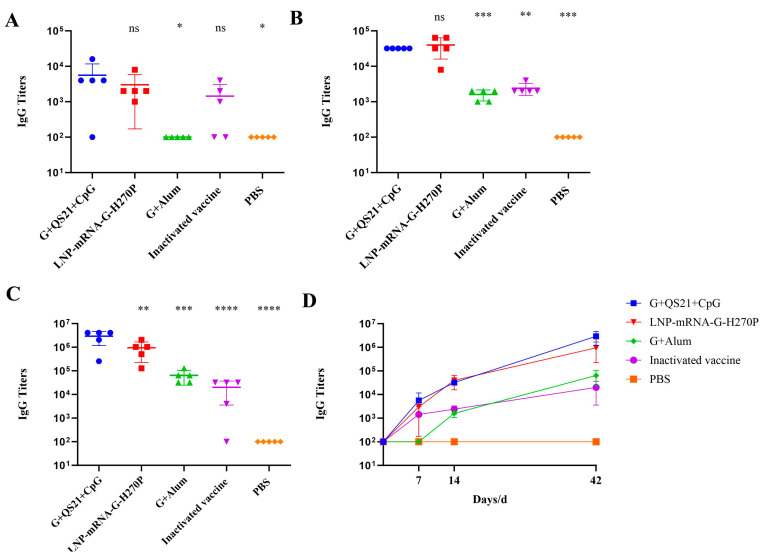
**The titers of RABV-G-specific IgG were detected by enzyme-linked immunosorbent assay (ELISA).** (**A**) IgG titers at 7 days post first immunization. (**B**) IgG titers at 14 days post first immunization. (**C**) IgG titers at 42 days post first immunization. (**D**) Changes in IgG titers before and after the first immunization over time. IgG titers were compared using one-way analysis of variance (ANOVA) followed by Dunnett’s multiple comparisons test, with the G+QS21+CpG vaccine group as a control. * *p* < 0.05. ** *p* < 0.01. *** *p* < 0.001. **** *p* < 0.0001. ns, no significant difference.

At day 7 after the first immunization, antigen-specific IgG antibody titers were rapidly induced in the G+QS21+CpG group (5620), LNP-mRNA-G-H270P group (3000), and inactivated vaccine group (1440), whereas no detectable IgG response was observed in the G+Alum vaccine group. Due to the low overall antibody levels at this early time point, no statistically significant differences were observed among groups (Figure 2A). By day 14, all vaccinated groups exhibited a substantial increase in IgG titers (Figure 2B). Notably, the LNP-mRNA-G-H270P group achieved the highest RABV-G-specific IgG titer (40,000), which was 1.25-fold higher than that of the G+QS21+CpG group (32,000; *p* = 0.5953), 25-fold higher than the G+Alum vaccine group (1600; *p* < 0.001), and 16.7-fold higher than the inactivated vaccine group (2400; *p*< 0.01). Prior to challenge, final antibody measurements revealed sustained high IgG levels in most vaccine groups (Figure 2C). The G+QS21+CpG group achieved the highest RABV-G-specific IgG titer (2,918,400), which was 3.08-fold higher than the LNP-mRNA-G-H270P group (947,200; *p* < 0.01), 45.6-fold higher than the G+Alum group (64,000; *p* < 0.001), and 145.77-fold higher than the inactivated vaccine group (20,020; *p* < 0.0001). Analysis of IgG dynamics revealed distinct immune response patterns among the groups (Figure 2D). Both the G+QS21+CpG and LNP-mRNA-G-H270P groups demonstrated rapid seroconversion, higher peak titers, and sustained antibody elevation over time. In contrast, the G+Alum vaccine group exhibited a delayed IgG response with lower overall titers, while the inactivated vaccine group showed early induction but plateaued at later time points.

**Figure 3:**
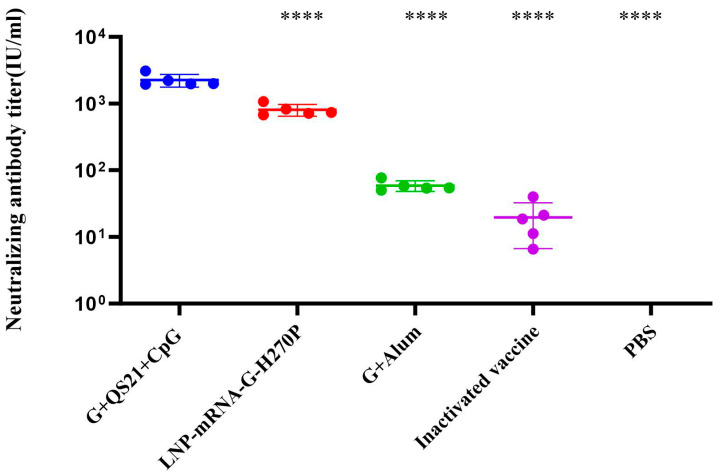
**RVNA levels were quantified using the RFFIT method.** Statistical analysis was performed by one-way ANOVA with Dunnett’s post-hoc test, using the G+QS21+CpG formulation as the reference group. **** *p* < 0.0001.

The RFFIT results revealed significant differences in RVNA titers among vaccination groups (Figure 3). The G+QS21+CpG group elicited the highest RVNA titer (2246 IU/mL), demonstrating 3-fold (806 IU/mL, *p* < 0.0001), 38-fold (59 IU/mL, *p* < 0.0001), and 115-fold (20 IU/mL, *p* < 0.0001) higher neutralizing activity compared to the LNP-mRNA-G-H270P group, G+Alum vaccine group, and inactivated vaccine groups, respectively. The PBS control group showed undetectable responses, confirming assay validity. This value substantially exceeds (by approximately 4500-fold) the internationally recognized protective threshold of 0.5 IU/mL established by the World Health Organization (WHO) for human rabies prophylaxis.

### 3.2. QS21+CpG Adjuvanted Vaccine Elicits Superior Cellular Immunity

**Figure 4:**
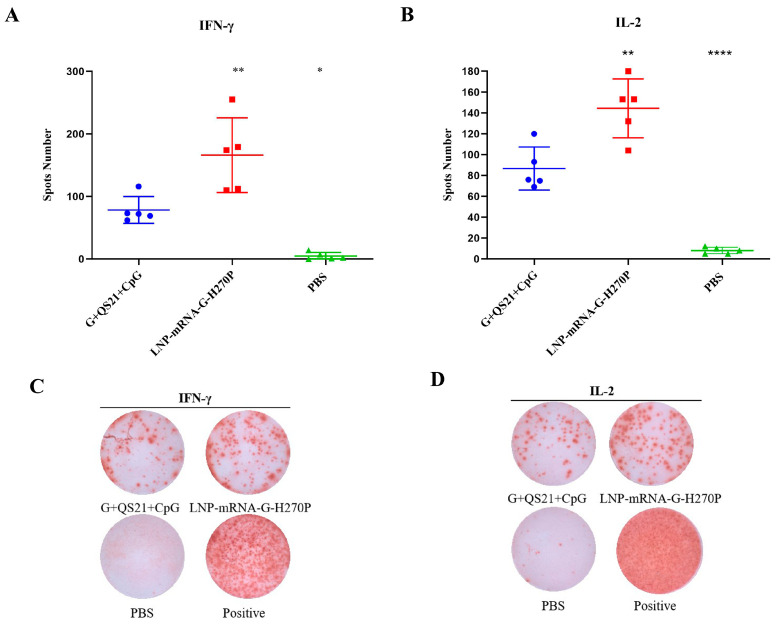
**ELISPOT analysis of splenocyte cytokine secretion profiles.** (**A**) RABV-G-stimulated IFN-γ secretion in splenocyte cultures. (**B**) IL-2 production by splenocytes following RABV-G stimulation. (**C**) Representative ELISPOT wells demonstrating IFN-γ-secreting cells. (**D**) Representative images of IL-2-producing splenocytes. ELISPOT numbers were compared using one-way ANOVA followed by Dunnett’s multiple comparisons test, with the G+QS21+CpG vaccine group as a control. * *p* < 0.05. ** *p* < 0.01. **** *p* < 0.0001.

**Figure 5:**
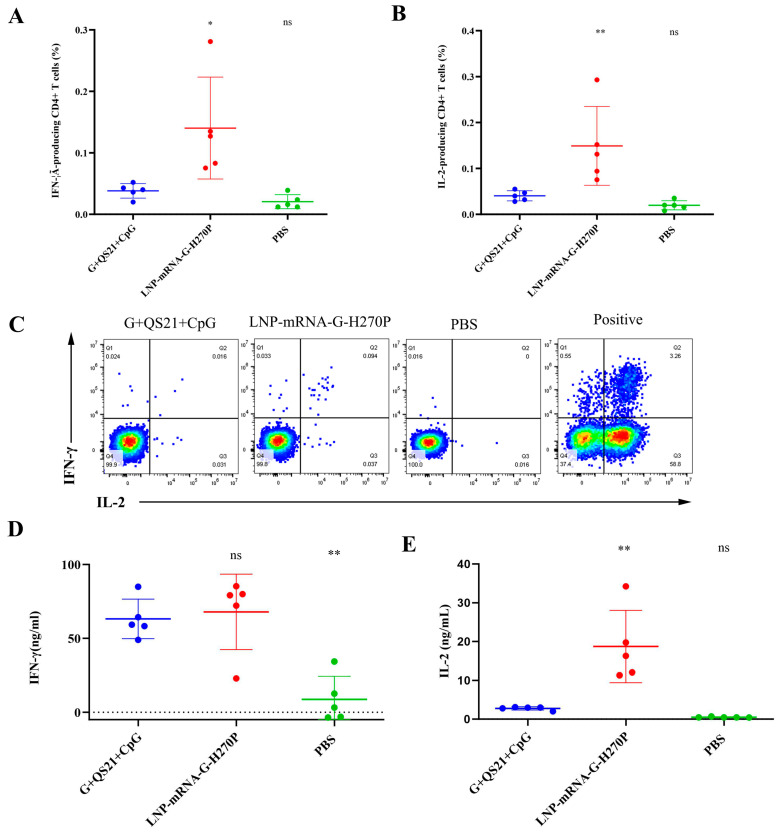
**Flow cytometric analysis of RABV-G-specific Th1 cytokine responses in CD4+ T cells.** (**A**) IFN-γ production by antigen-stimulated CD4+ T cells. (**B**) IL-2-secreting CD4+ T cell population following RABV-G stimulation. (**C**) Pseudocolor images provide a visual representation of the average expression levels of IFN-γ (Q1+Q2) and IL-2 (Q3+Q2) in a specific CD4+ T cell population. ELISA was used to measure the concentrations of IFN-γ (**D**) and IL-2 (**E**) secreted by splenocytes upon stimulation with 10 μg/mL RABV-G. Data were compared using one-way ANOVA followed by Dunnett’s multiple comparisons test, with the G+QS21+CpG vaccine group as a control. * *p* < 0.05. ** *p* < 0.01. ns, no significant difference.

The ELISPOT results analysis indicated that the G+QS21+CpG group induced 78 spots upon RABV-G stimulation, representing a significant 15.6-fold increase over the PBS group (5 spots, *p* < 0.05). The LNP-mRNA-G-H270P group (166 spots per 3 × 10^5^ splenocytes) demonstrated a higher number of IFN-γ-secreting splenocytes compared to the G+QS21+CpG group (Figure 4A). With regard to RABV-G -specific IL-2-secreting splenocytes (Figure 4B), the G+QS21+CpG group elicited 87 spots, significantly higher than the PBS group (eight spots, 10.88-fold lower, *p* < 0.0001). In comparison, the LNP-mRNA-G-H270P group showed a further 1.66-fold increase (144 spots) over the G+QS21+CpG group (*p* < 0.01), both of which were found to be statistically significant. In duplicate independent experiments, splenocytes from the G+QS21+CpG group and the LNP-mRNA-G-H270P group generated significantly higher numbers of cytokine-secreting cells, as reflected by dense, well-defined spot formations (Figure 4C,D), compared to the PBS group. The qualitative spot patterns were consistent with the quantitative spot-forming cell (SFC) enumeration results (Figure 5A,B), further validating the enhanced cellular immune responses in the G+QS21+CpG group and LNP-mRNA-G-H270P group.

Flow cytometry analysis yielded findings consistent with the ELISPOT results. After stimulation with RABV-G, the flow cytometry analysis revealed that the G+QS21+CpG group showed 0.039% of CD4+ T cells producing IFN-γ (Figure 5A), representing a significant 1.95-fold increase over the PBS group (0.020%, *p* = 0.7949). The LNP-mRNA-G-H270P group demonstrated superior cellular immunity, with IFN-γ-producing CD4+ T cells reaching 0.140%, representing a 3.59-fold increase compared to the G+QS21+CpG benchmark (*p* < 0.05). Similarly, for IL-2 expression (Figure 5B), the G+QS21+CpG group induced responses in 0.040% of CD4+ T cells (2.11-fold higher than PBS at 0.019%, *p* = 0.7459). The LNP-mRNA-G-H270P formulation again showed the stronger performance (0.149%), achieving 3.7-fold greater IL-2 response than G+QS21+CpG (*p* < 0.01). All comparisons showed statistically significant differences. Figure 5C summarizes the cytokine profile, confirming that the vaccine formulation (G+QS21+CpG group and LNP-mRNA-G-H270P group) not only enhances IFN-γ production but also promotes IL-2 secretion, indicative of a polyfunctional T cell response. Positive controls exhibited robust signal responses, validating the reliability of the experimental system. Meanwhile, the PBS group showed negligible background reactivity, effectively excluding nonspecific interference.

Following RABV-G stimulation, splenocyte cultures from the LNP-mRNA-G-H270P group demonstrated significantly enhanced cytokine production (Figure 5). The IFN-γ secretion reached 69.31 ng/mL, representing a 1.11-fold increase over the G+QS21+CpG group (62.56 ng/mL, *p* = 0.893) and a 5.63-fold elevation compared to PBS controls (12.32 ng/mL, *p* < 0.001). More strikingly, IL-2 levels in the LNP-mRNA-G-H270P group (19.51 ng/mL) exceeded those in the G+QS21+CpG group by 6.0-fold (3.25 ng/mL; *p* < 0.0001) and surpassed PBS group levels by 39.82-fold (0.49 ng/mL; *p* < 0.0001).

After stimulation with RABV-G, cytokine analysis revealed significant immunological differences between groups. The G+QS21+CpG group demonstrated cytokine production with IFN-γ levels of 63.13 ng/mL, significantly higher (7.25-fold, *p* < 0.01) than the PBS control (8.71 ng/mL). The LNP-mRNA-G-H270P formulation showed modest enhancement in IFN-γ secretion (67.89 ng/mL, 1.08-fold vs. G+QS21+CpG, *p* = 0.8929). More strikingly, IL-2 production showed dramatic increases, with the G+QS21+CpG group (2.78 ng/mL) already exhibiting 5.67-fold higher levels than PBS (0.49 ng/mL, *p* = 0.7345), while the LNP-mRNA-G-H270P group achieved 18.71 ng/mL, representing a 6.73-fold improvement over the G+QS21+CpG benchmark (*p* < 0.01) (Figure 5D,E).

### 3.3. Protective Efficacy Against Lethal Challenge in Mice

**Figure 6:**
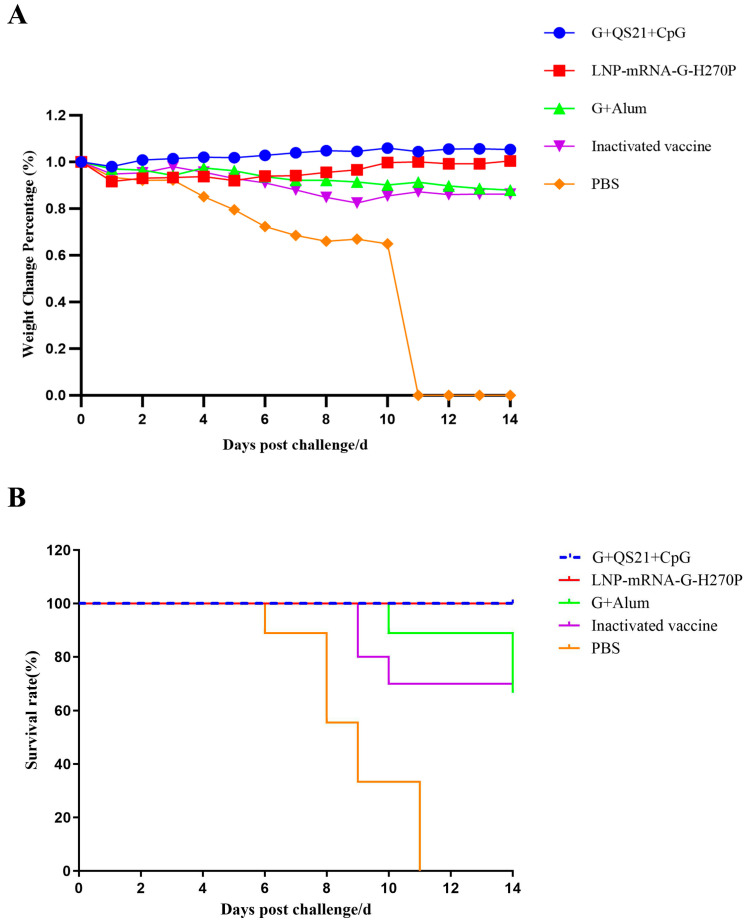
**Body weight changes and survival rate in mice after virus challenge.** (**A**) Mice daily body weight percentage change relative to the initial body mass were calculated over 14 days post-infection. (**B**) Kaplan–Meier survival curves showing the percentage of surviving mice over 14 days post-infection. Mice were inoculated with CVS-11 at a dose of 50LD50/mouse (*n* = 10/group).

Following immunization and subsequent lethal challenge with 50LD_50_ of CVS-11 strain, all vaccine groups exhibited significantly improved outcomes compared to the PBS group (Figure 6). Body weight dynamics (Figure 6A) revealed stable maintenance in the G+QS21+CpG and LNP-mRNA-G-H270P groups throughout the 14-day observation period, indicating minimal clinical impact of infection. In contrast, the G+Alum vaccine group and inactivated vaccine groups showed transient weight loss, though all surviving animals regained baseline weight by day 10.

Survival analysis (Figure 6B) demonstrated 100% protection in both G+QS21+CpG and LNP-mRNA-G-H270P groups, with the G+QS21+CpG group showing faster recovery of physiological parameters. The G+Alum vaccine group demonstrated a 70% survival rate post-challenge, equivalent to the protection efficacy observed in the inactivated vaccine positive control group. All PBS control mice succumbed to infection by day 11, confirming challenge validity. Notably, the weight–survival correlation indicated that the G+QS21+CpG and LNP-mRNA-G-H270P groups not only prevented mortality but also maintained optimal health status, as evidenced by the absence of significant weight fluctuation.

## 4. Discussion

Subunit vaccines have emerged as a well-established vaccine platform with distinct advantages in safety and manufacturability [23]. By utilizing purified antigenic components, this approach elicits highly specific immune responses while eliminating potential risks associated with pathogen inactivation procedures. The inherent stability and simplified storage requirements of subunit vaccines, coupled with their scalable production capacity, make them particularly suitable for global immunization programs, especially in resource-limited settings [24]. While subunit vaccines may induce relatively narrower immune responses compared to mRNA-based platforms, this limitation can be effectively addressed through rational adjuvant selection [25]. The QS21+CpG adjuvant system has demonstrated particular promise in enhancing immunogenicity while maintaining a balanced humoral and cellular immune profile [26,27]. This balanced immune activation is especially critical for rabies prevention, where both antibody-mediated neutralization and T cell responses play essential roles in viral clearance and long-term protection [28].

The QS21+CpG-adjuvanted RABV-G subunit vaccine developed in this study demonstrated superior immunogenicity compared to other vaccine platforms. Notably, it induced significantly higher levels of G protein-specific IgG antibodies and virus-neutralizing titers than both the H270P-mutated mRNA vaccine and conventional vaccine groups. This robust humoral immune response is particularly valuable for PEP, where rapid seroconversion is critical for effective protection against rabies infection [29]. While the mRNA vaccine platform showed stronger cellular immune responses, as evidenced by 3.64-fold higher antigen-specific CD4+ T cell activation compared to the subunit vaccine, both platforms achieved complete protection against lethal challenge. This finding suggests that once neutralizing antibody titers exceed a critical threshold, complete protection can be attained regardless of cellular immune magnitude [30]. The substantial Th1 response induced by QS21+CpG, though less potent than mRNA vaccination, appears sufficient for protective immunity [17].

The distinct immune mechanisms underlying vaccine efficacy involve two primary pathways: humoral immunity mediated by antibody-dependent viral neutralization and pathogen clearance, and cellular immunity driven by T cell-mediated elimination of infected cells [31]. The superior humoral responses elicited by the QS21+CpG-adjuvanted RABV-G subunit vaccine can be attributed to its unique immunomodulatory properties. QS21, a triterpenoid saponin derivative, exhibits dual functionality by directly activating B cells while concurrently promoting dendritic cell maturation and antigen presentation capacity [8]. This is complemented by CpG oligodeoxynucleotides which engage TLR9 signaling to enhance B cell differentiation into antibody-secreting plasma cells and prolong their survival—accounting for the sustained antibody titers observed in our studies [2]. The adjuvant combination establishes an immunologically favorable microenvironment through two synergistic mechanisms: QS21-mediated antigen retention creates local depots, while CpG induces a pro-inflammatory milieu that enhances lymphocyte recruitment and activation [32]. The synergistic combination of QS21 and CpG induces balanced Th1/Th2 polarization, eliciting robust cellular immunity while maintaining potent humoral responses. Through coordinated activation of multiple immune cell populations, this adjuvant system significantly enhances both antibody titers and neutralizing capacity, resulting in superior humoral immune performance [16].

Previous work demonstrated that the H270P-mutated mRNA vaccine enhances proteasomal processing and cross-presentation of antigens, leading to robust CD4+ T cell activation and subsequent elimination of infected cells [6,33]. In contrast, while the QS21+CpG-adjuvanted subunit vaccine elicits measurable Th1 responses, its capacity to activate CD4+ T cells remains comparatively limited [17]. This difference primarily stems from the adjuvant system’s preferential activation of APCs and B cells to enhance humoral immunity, with relatively modest direct effects on cytotoxic T cell stimulation.

The observed reductions in IFN-γ and IL-2 production induced in the G+QS21+CpG group compared to mRNA vaccination likely reflects the superior intracellular antigen expression and presentation efficiency inherent to mRNA platforms [34]. These mechanistic insights provide a rational explanation for the observed differences in cellular immune responses between vaccine formulations. Importantly, these findings not only validate the utility of the QS21+CpG adjuvant system for rabies subunit vaccines, but also provide important insights for optimizing vaccine design strategies based on desired immune outcomes.

## 5. Conclusions

In conclusion, our QS21+CpG-adjuvanted RABV-G subunit vaccine demonstrates superior immunogenicity, combined with the rapid response kinetics of mRNA vaccines. In addition, injection of two doses of this vaccine can resist a lethal dose attack of the CVS-11 strain. These characteristics underscore the strong translational potential of this vaccine platform, warranting further development as a next-generation rabies prophylactic candidate. However, difficulties with yield and purification of the recombinant RABV-G protein, as well as the cost of adjuvant QS21, may affect the final application of the vaccine.

## Figures and Tables

**Table 1:** Overview of composition and dosage of each vaccine group.

Vaccine Group	mRNA (μg/dose)	RABV-G (μg/dose)	QS21 (μg/dose)	CpG (μg/dose)	Alum (mg/dose)	Inactivated Virus (≥2.5 IU/dose)
G+QS21+CpG	-	5	5	10	-	-
LNP-mRNA-G-H270P	15.4	-	-	-	-	-
G+Alum	-	5	-	-	1	-
Inactivated vaccine	-	-	-	-	-	1/6
PBS	-	-	-	-	-	-

- Not added.

## Data Availability

All data used during the study are available from the corresponding author by request.

## Supplementary Materials

The following supporting information can be downloaded at: https://www.mdpi.com/article/10.3390/vaccines13080887/s1, Figure S1: Characterization of LNP-mRNA-G-H270P vaccine. Table S1. Detailed weight data in Figure 6A.
