# Supplementary material for: A QS21+ CpG-Adjuvanted Rabies Virus G Subunit Vaccine Elicits Superior Humoral and Moderate Cellular Immunity
Source: Vaccines (Basel). 2025 Aug 21;13(8):887. doi: 10.3390/vaccines13080887 (PMC12390428; doi:10.3390/vaccines13080887)
Supplement: Supplementary file 1 [file vaccines-13-00887-s001.zip › vaccines-3795197-supplementary.pdf]

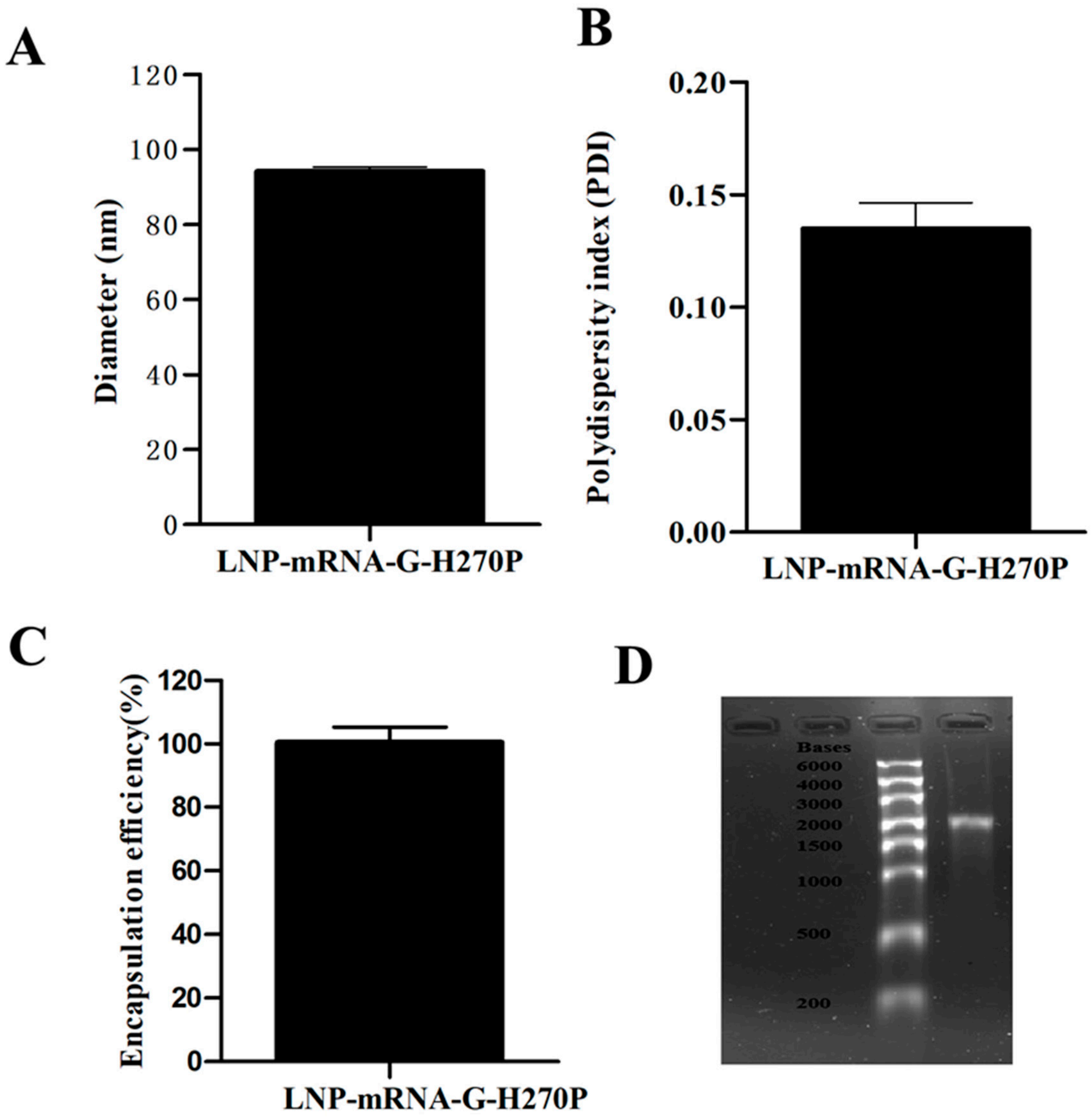

Figure S1. Characterization of LNP-mRNA-G-H270P vaccine. (A) Diameter of the LNP-mRNA-G-H270P vaccine; (B) Polydispersity index (PDI) of LNP-mRNA-G-H270P vaccine; (C) Encapsulation efficiency of LNP-mRNA-G-H270P vaccine; (D) 1% denatured agarose gel electrophoresis to determine the integrity of LNP-mRNA-G-H270P vaccine.

Table S1. Detailed weight data in Fig 6A.

	LNP-mRNA-G-H270P		G+QS21+CpG		G+Alum		Inactivated vaccine		PBS	
days	Weight Change Percentage (%)	average value	Weight Change Percentage (%)	average value	Weight Change Percentage (%)	average value	Weight Change Percentage (%)	average value	Weight Change Percentage (%)	average value
0	1.00	22.25	1.00	21.79	1.00	21.87	1.00	22.27	1.00	21.92
1	0.92	20.37	0.98	21.36	0.97	21.22	0.95	21.14	0.93	20.48
2	0.93	20.70	1.01	21.96	0.96	21.10	0.95	21.24	0.92	20.19
3	0.93	20.77	1.02	22.12	0.94	20.62	0.98	21.80	0.92	20.21
4	0.94	20.87	1.02	22.24	0.97	21.30	0.96	21.32	0.85	18.66
5	0.92	20.46	1.02	22.19	0.96	21.03	0.93	20.68	0.80	17.43
6	0.94	20.92	1.03	22.39	0.94	20.49	0.91	20.26	0.72	15.86
7	0.94	20.95	1.04	22.63	0.92	20.13	0.88	19.60	0.68	15.01
8	0.96	21.26	1.05	22.83	0.92	20.14	0.85	18.89	0.66	14.46
9	0.97	21.50	1.04	22.77	0.91	20.00	0.82	18.37	0.67	14.66
10	1.00	22.19	1.06	23.07	0.90	19.70	0.85	19.03	0.65	14.23
11	1.00	22.28	1.04	22.74	0.91	19.98	0.87	19.43	0.00	0.00
12	0.99	22.08	1.06	22.99	0.90	19.63	0.86	19.14	0.00	0.00
13	0.99	22.08	1.06	23.01	0.89	19.38	0.86	19.19	0.00	0.00
14	1.00	22.34	1.05	22.95	0.88	19.23	0.86	19.20	0.00	0.00
